# The Effect of Date Seed (*Phoenix dactylifera*) Extract on Paraoxonase and Arylesterase Activities in Hypercholesterolemic Rats

**DOI:** 10.17795/jjnpp-10368

**Published:** 2014-02-15

**Authors:** Mohammad Reza Takaeidi, Alireza Jahangiri, Mohammad Javad Khodayar, Amir Siahpoosh, Hamid Yaghooti, Saeid Rezaei, Maryam Salecheh, Zahra Mansourzadeh

**Affiliations:** 1 Medicinal Plants and Natural Products Research Center, School of Pharmacy, Ahvaz Jundishapur University of Medical Sciences, Ahvaz, IR Iran; 2 Department of Medicinal Chemistry and Nanotechnology center, School of Pharmacy, Ahvaz Jundishapur University of Medical Sciences, Ahvaz, IR Iran; 3 Department of Pharmacology and Toxicology Research Center, School of Pharmacy, Ahvaz Jundishapur University of Medical Sciences, Ahvaz, IR Iran; 4 Department of Pharmacognosy, School of Pharmacy, Ahvaz Jundishapur University of Medical Sciences, Ahvaz, IR Iran; 5 Department of Medical Laboratory Sciences, School of Allied Medical Sciences, Ahvaz Jundishapur University of Medical Sciences, Ahvaz, IR Iran; 6 Department of Pharmaceutics, School of Pharmacy, Zanjan University of Medical Sciences, Zanjan, IR Iran

**Keywords:** Arylesterase, Aryldialkylphosphatase, Antioxidants, Rats

## Abstract

**Background:**

Paraoxonase 1 (PON1) is a high- density lipoprotein (HDL)-associated enzyme, displaying esterase and lactonase activity. The PON1 is involved in a variety of inflammatory diseases, metabolizing toxic oxidized lipids and detoxifying of organophosphorus insecticide compounds and nerve agents.

**Objectives:**

The aim of this study was to investigate the effects of methanolic date seed extract (DSE) on paraoxonase and arylesterase activities in hypercholesterolemic rats.

**Materials and Methods:**

Experiments were conducted in two groups of normal and hypercholesterolemic rats and continued for four weeks. Two weeks after receiving the normal and hypercholesterolemic diet, different dosages of DSE were administered during the last two weeks of the treatment. Blood samples were taken from animals before administration of DSE (at day 14) and at the end of the experimental period (at day 28). Paraoxonase and arylesterase activities of PON1 enzyme were assayed by kit using paraoxone and phenylacetate as the substrates. Relative changes in serum paraoxonase and arylesterase activities and total antioxidant capacity (TAOC) were compared between the two groups during this interval.

**Results:**

Administration of DSE significantly increased serum paraoxonase and arylesterase activities in treated hypercholesterolemic groups compared to untreated ones. There was a significant difference in the TAOC of serum between the normal diet and hypercholesterolemic groups. However, DSE did not change the TAOC in hypercholesterolemic groups significantly.

**Conclusions:**

DSE increases serum paraoxonase and arylesterase activities. These beneficial effects may be subjected to the presence of natural antioxidants such as phenolic compounds in the date seed. Despite this, DSE did not increase TAOC in treated hypercholesterolemic groups compared to the untreated ones based on ABTS (2,2'-azino-di-(3-ethylbenzothiazoline)-6-sulfonic acid) radical reduction assay. This indicates that the hypercholesterolemic diet, apart from DSE and atorvastatin effects, may be responsible for the serum TAOC reduction. However, it is concluded that DSE may be useful in decreasing the symptoms of diseases resulting from the low activity of paraoxonase.

## 1. Background

Three-gene family of PON was detected and evaluated, comprising different properties. PON1 is an antioxidant and a high density lipoprotein (HDL) associated enzyme which is synthesized in the liver. PON1 displays arylesterase activity and phenyl acetate is one of its best substrates. PON1 shows organophosphatase activity explaining its ability of hydrolyzing organophosphorous insecticides. Furthermore, PON1 has good lactonase activity, by which it can hydrolyze a wide range of lactones; and another capability of PON1 is the homocysteine thiolactonase activity (1). For the first time, PON1 was diagnosed for its ability of detoxifying organophosphate compounds, so it was named paraoxonase (2). PON1 metabolizes toxic oxidized lipids of low density lipoproteins (LDLs) and HDLs, and hydrolyzes several organophosphorus (OP) insecticides, nerve agents, some of drugs and endogenous lactones (2-4). With regards to the important role of this enzyme, increase of PON1 activity or expression may be protective against oxidative stress and the acute toxicity of certain OP insecticides. This activity elevation may be useful in reduced PON1 activity in diabetes, atherosclerosis and other cardiovascular diseases (5-7). Many studies have been accomplished about the administration of drugs and natural dietary products to increase the activity or expression of PON1 (2). PON1 activity can get increased by statin drugs (8), anti-diabetic drugs such as sulphonylureas (9) , rosiglitazone (10), dietary polyphenols (11, 12) and grape seed extract (12). It has been reported that PON1 is an antidiabetic enzyme (11) that increases the insulin release from pancreatic beta cells (13). *Phoenix dactylifera* L., generally called the date palm, is a valuable plant that grows is grown in the Southwest Asia and North Africa. Based on the phytochemical studies, date fruits contain anthocyanins, phenolics, sterols, carotenoids, procyanidins and flavonoids (14). These natural compounds are known to function as free radical scavenger, antioxidant, antimutagenic, anti-inflammatory, hepatoprotective and nephroprotective agents (14). Seeds of date palm (pits) are waste products of many industries that are added to the domesticated animals foods after technological alteration in the date fruits. Investigations suggest that the date seeds are free from any toxic effects. Date seeds are rich in protein (5.1 g/100 g), fat (9.0 g/100 g), dietary fiber (73.1 g/100 g), phenolics (3942 mg/100 g), and antioxidants (14, 15). Although the date palm fruit served as the low cost food for millions of people around the world for several centuries, studies on its well-being benefits are inadequate and its hardly recognized as a healthy food by the health professionals and the public (16). It has been reported that the total phenolic content of seeds of several fruits such as mango, avocado, and jackfruit, was higher than their edible flesh (17). Date seeds can also be considered as a valuable source of phenolic compounds and an inexpensive rich source of natural dietary fiber and antioxidants (18, 19). Regarding the presence of antioxidants such as phenolic compounds in the date seed which is potential to scavenge free radicals (14, 17) as well as the impact of natural antioxidant and PON1 activity, it can be concluded that DSE may be effective on PON1 activity. However, in response to protection against atherosclerosis development and overcoming toxicity of certain OP, the present study was designed to investigate DSE on serum paraoxonase and arylesterase activities and antioxidant capacity.

## 2. Objectives

The aim of this study was to investigate the effects of methanolic date seed extract (DSE) on paraoxonase and arylesterase activities in hypercholesterolemic rats.

## 3. Materials and Methods

### 3.1. Plant Materials

The date fruits were collected during the last stage of ripening process in which the dates look dehydrated (Tamar stage), from Behbehan, Iran, and identified by Dr. Siahpoosh (Department of Pharmacognosy and Medicinal Plant Research Center, Jundishapur School of Pharmacy, Ahvaz). Seeds were isolated from fruits, then soaked in water and washed to remove any adhered date ﬂesh. The seeds were air-dried under the shade at room temperature and grinded to convert the seeds to powder.

### 3.2. Preparation of Extract

Powdered date seeds were extracted after 72 hours of maceration in methanol. The extract was then concentrated under reduced pressure in a rota evaporator to reach the desired volume. The solvent was removed using freeze dryer (operon). The yield of the extract (dry powder) was calculated to be 4.2%.

### 3.3. Animals

Male NMRI rat weighing 200 ± 20 gram was obtained from the experimental animal house of Ahvaz University of Medical Sciences. All animals were maintained under controlled conditions of 25 ± 2˚C and 12 hours light-dark cycles with free access to food and drinking water except during the experiments. All the ethical issues were considered based on the Ahvaz Medical University Ethical Protocols (AMUEP) in animal experiments.

### 3.4. Drug, Diet and Kit

Atorvastatin pure powder (Exir Pharmaceutical Manufacturing Company, Borujerd, Iran), cholesterol and cholic acid (Merck, Germany) were used. Paraoxonase, arylesterase and antioxidant activity were measured using commercially available kits (Rel Assay Diagnostics®, MEGA TIP San, Turkey). DSE and atorvastatin were diluted in distilled water and administered orally in a volume of 2 mL/kg/d. DSE and atorvastatin solutions were prepared immediately before use. Hypercholesterolemic diet contains 98.5% normal diet, 1% cholesterol and 0.5% cholic acid.

### 3.5. Experimental Procedure

At first, experiments were conducted in two groups of normal and hypercholesterolemic rats. Two weeks later, the normal diet group was divided into three sub-groups including control, atorvastatin and high SDE dosage (1000 mg/kg), and the hypercholesterolemic group was divided into five sub-groups including control, atorvastatin and those treated with different dosages of SDE (250, 500 and 1000 mg/kg). Different dosages of DSE were administered in normal and hypercholesterolemic rats during the two successive weeks. Blood samples were collected in centrifuge tubes and centrifuged to obtain plasma at days 14 and 28. The serum samples were stored in -20˚C to be analyzed with microplate reader (Sunrise Tecan microplate reader).

### 3.6. Paraoxonase Activity Assay

Paraoxonase activity of PON1 was measured by kit. The kit consists of substrate solution and Tris buffer. The substrate solution comprising paraoxone and buffer solution contains calcium ion as a cofactor of PON1 enzyme. Activity assay was performed according to the kit instruction. Hydrolysis of paraoxone in the sample produces p-nitrophenol, absorbance linear increase of which at wavelength of 412 nm was considered as enzyme activity. The molar absorptivity of p-nitrophenol is 18,290 M^-1^ cm^-1^ and one unit of paraoxonase activity equals 1 µmol of paraoxon hydrolyzed per Liter per minute at 37˚C.

### 3.7. Arylesterase Activity Assay

Arylesterase activity was assayed according to the kit. The substrate solution contains phenyl acetate hydrolysis of which yields phenol and acetic acid. The produced phenol is measured by plate reader through oxidative coupling with 4-aminoantipyrine and potassium ferricyanide. Serum dilution ratio for arylesterase activity was 1/100 (serum/diluent). The molar absorptivity of colored complex is 4000 M^-1^ cm^-1^ and one unit of arylesterase activity equals 1 mmol of phenol produced per liter per minute at 37˚C. 

### 3.8. Total Antioxidant Capacity Assay

Antioxidants in the sample reduce dark blue-green colored ABTS radical to colorless ABTS form. The absorbance change at 660 nm is related to the total antioxidant level of the sample. The assay is performed with standard 1 (0.0 mmol Trolox Equivalent/L) solution and antioxidant standard solution (St2) which is traditionally named as Trolox Equivalent (1.0 mmol Trolox Equivalent/L). The △absorption for each of them is calculated based on the difference of absorption in the presence of ABTS with its absence. Total antioxidant capacity is calculated as below:

Antioxidant Capacity = ∆Absorbance St1 - ∆Absorbance Sample / ∆Absorbance St1 - ∆Absorbance St2

### 3.9. Statistical Analysis

The ratio of mean differences in serum paraoxonase, arylesterase activities and TAOC between days 28 and 14 to day 14 were calculated and reported as follows. Analysis of variance (ANOVAs) followed by Tukey’s test were used for comparison of the data. Differences between means were considered statistically significant (P < 0.05). Each point is expressed as Mean (SEM) for six rats.

Relative Change, % = (Mean value at day 28 – Mean value at day 14 / Mean value at day 14) × 100

## 4. Results

### 4.1. The Effects of DSE on the Serum Paraoxonase Activity

**Figure 1.**
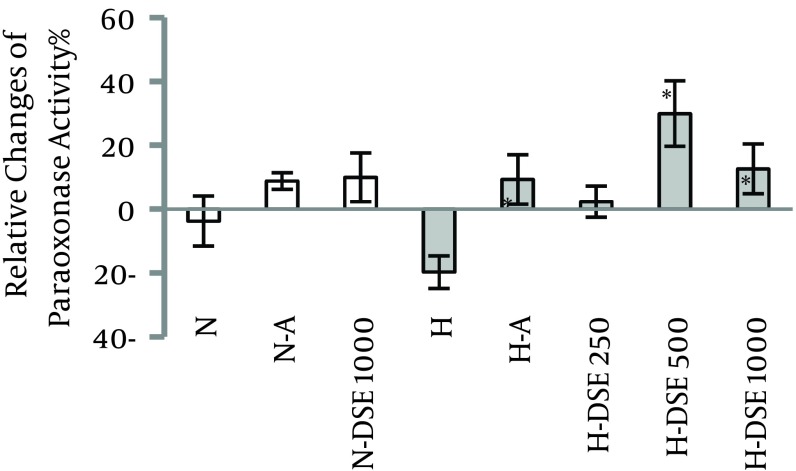
The Effects of A (Atorvastatin (10 mg/kg/d) and DSE (Date Seed Extract (250, 500 and 1000 mg/kg/d) on Percentage of Relative Change of Paraoxonase Activity on N (Normal) and H (Hypercholesterolemic) Rats Data are expressed as mean ± SEM. * Statistical significant differences compared to the hypercholesterolemic (H) group at P < 0.05.

In hypercholesterolemic groups, administration of DSE at the doses of 500 and 1000 mg/kg and atorvastatin significantly increased serum paraoxonase activity compared to the untreated group. The effect of extract was dose-dependent. However, these effects were not significant between normal diet and hypercholesterolemic groups (Figure 1). 

### 4.2. The Effects of DSE on the Serum Arylesterase Activity

**Figure 2.**
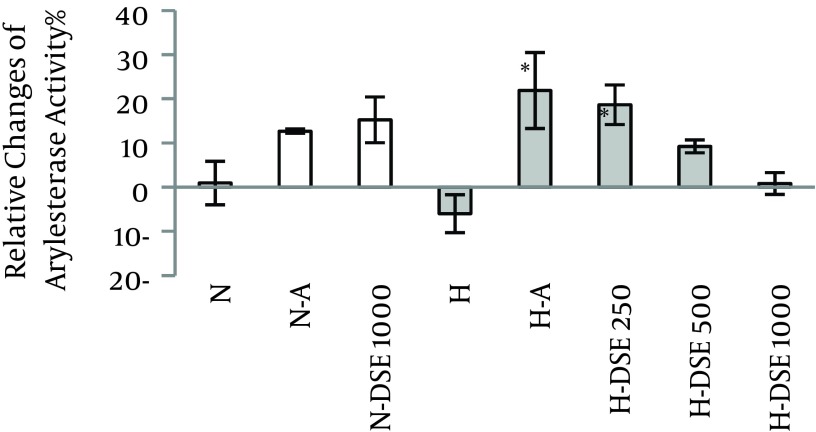
The Effect of A (Atorvastatin (10 mg/ kg/d) and DSE (Date Seed Extract (250, 500 and 1000 mg/kg/d) on the Percentage of Relative Change of Arylesterase Activity on N (Normal) and H (Hypercholesterolemic) Rats Data are expressed as mean ± SEM. *Statistical significant differences compared to the hypercholesterolemic (H) group at P < 0.05.

Administration of DSE at dosage of 250 mg/kg significantly increased the serum arylesterase activity in treated hypercholesterolemic groups compared to the untreated ones. However, this effect was not significant between normal and hypercholesterolemic groups (Figure 2). 

### 4.3. The Effects of DSE on the Total Antioxidant Capacity

**Figure 3.**
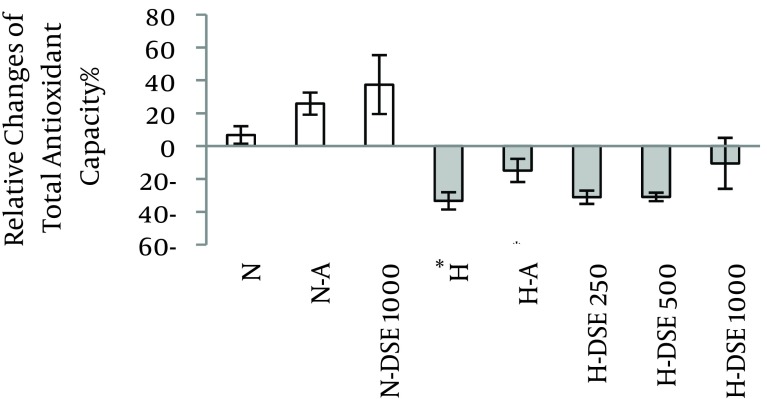
The effect of A (Atorvastatin (10 mg/ kg/d) and DSE (Date Seed Extract (250, 500 and 1000 mg/kg/d) on the Percentage of Relative Change of Total Antioxidant Capacity on N (Normal) and H (Hypercholesterolemic) Rats Data are expressed as mean ± SEM. * Statistical significant differences compared to the normal diet respective group at P < 0.05.

Hypercholesterolemic diet significantly decreased the TAOC of serum. Administration of DSE at any dosage as well as atorvastatin did not reverse the decreased TAOC of serum in the hypercholesterolemic group (Figure 3). 

## 5. Discussion

It is obvious that high total cholesterol level is an important risk factor for development and progression of atherosclerosis. Lipoprotein oxidation is one of the basic mechanisms involved in the initiation and progression of atherosclerosis. The present study was designed to investigate the normal-diet and hypercholesterolemic rats. DSE increased paraoxonase and arylesterase activity of serum in hypercholesterolemic rats. These effects may be related to the presence of natural antioxidants such as phenolic compounds in the date seed. Date seeds are known as important sources of phenolic acids consisting of hydroxylated derivatives of benzoic acid (gallic acid, protocatechuic acid, p-hydroxybenzoic acid and vanillic acid) and cinnamic acid (caffeic acid, p-coumaric acid, ferulic acid, m-coumaric and o-coumaric acid) which possess antioxidant effects (18). These antioxidant compounds may be responsible for the increased activity of paraoxonase through direct interaction with enzyme or stabilization of paraoxonase on HDL and/or its expression. There are evidences suggesting that PON1 is an HDL-associated antioxidant enzyme protecting against atherosclerosis by preventing from lipoprotein oxidation and hydrolyzing oxidized cholesterol and/or phospholipids in atherosclerotic lesions and oxidized LDL (1). PON1 is readily inactivated by exogenous or endogenous oxidant agents (20). Therefore, dietary antioxidants are found to increase PON1 activity/expression in animals or humans (1). Many studies have revealed the reduction of paraoxonase enzyme activity in different situations, resulting in diseases such as oxidative stress, diabetes and cardiovascular importantly coronary artery disease. Reduction in paraoxonase activity of serum may be related to the elevation of lipid peroxide levels (1, 21). Atorvastatin that inhibits the cholesterol synthesis by inhibiting the enzyme (3-hydroxy-3-methylglutaryl-coenzyme A) HMG-CoA reductase, is reported to increase the serum paraoxonase activity (22, 23). On the other hand, other studies failed to find any changes in the serum paraoxonase activity under statin administration (24). Nevertheless, increased paraoxonase and arylesterase activity or DSE did not increase the TAOC in treated hypercholesterolemic groups compared to the untreated ones based on the ABTS radical reduction assay. However, TAOC in hypercholesterolemic groups was significantly lower than the respective normal groups. These indicate that the hypercholesterolemic diet, apart from the DSE effects and atorvastatin efficacy, is responsible for the serum TAOC reduction. In conclusion, these results demonstrated that DSE significantly increased the serum paraoxonase and arylesterase activity in hypercholesterolemic rats and DSE may be useful in decreasing symptoms of diseases related to the low activity of paraoxonase or overcoming diseases due to the increasing activity of paraoxonase such as OP poisoning.
